# Evolution of the FGF Gene Family

**DOI:** 10.1155/2012/298147

**Published:** 2012-08-07

**Authors:** Silvan Oulion, Stephanie Bertrand, Hector Escriva

**Affiliations:** CNRS, UMR 7232, BIOM, Université Pierre et Marie Curie Paris 06, Observatoire Océanologique, 66650 Banyuls-sur-Mer, France

## Abstract

Fibroblast Growth Factors (FGFs) are small proteins generally secreted, acting through binding to transmembrane tyrosine kinase receptors (FGFRs). Activation of FGFRs triggers several cytoplasmic cascades leading to the modification of cell behavior. FGFs play critical roles in a variety of developmental and physiological processes. Since their discovery in mammals, FGFs have been found in many metazoans and some arthropod viruses. Efforts have been previously made to decipher the evolutionary history of this family but conclusions were limited due to a poor taxonomic coverage. We took advantage of the availability of many new sequences from diverse metazoan lineages to further explore the possible evolutionary scenarios explaining the diversity of the FGF gene family. Our analyses, based on phylogenetics and synteny conservation approaches, allow us to propose a new classification of FGF genes into eight subfamilies, and to draw hypotheses for the evolutionary events leading to the present diversity of this gene family.

## 1. Introduction

Fibroblast growth factors (FGFs) form a family of generally extracellular signaling peptides, which are key regulators of many biological processes ranging from cell proliferation to the control of embryonic development in metazoans. Ever since the mitogenic activity of FGF-like factors was first observed in 1939 [1] and the first FGF factor was isolated in the 1970s [2], a large number of members of this gene family have been isolated and characterized in different metazoans.

FGFs are small proteins (between 17 and 34 kDa) characterized by a relatively well conserved central domain of 120 to 130 amino acids. This domain is organized into 12 antiparallel *β* sheets forming a triangular structure called beta trefoil. In general, FGFs function through binding to a tyrosine kinase receptor (FGFR) on the surface of the cell membrane. Two FGF ligands bind a dimeric receptor in the presence of heparan sulphate proteoglycan (HSPG) allowing the transphosphorylation and activation of the intracellular tyrosine kinase domain of the receptor. Binding to FGFRs usually activates several intracellular cascades (i.e., Ras/MAPK, PI3K/Akt, and PLC*γ*/PKC) which may regulate the transcription of different target genes. Through the activation of these cytoplasmic pathways, the FGF signal controls several major cellular functions such as cell proliferation, migration, differentiation, or survival. An intracellular mode of action has also been described in the case of FGF1 but it is poorly documented [3].

Concerning the evolutionary history of the FGF gene family, several studies using molecular phylogenetics as well as synteny conservation analyses have been performed [4–8]. The first phylogeny-based classifications of the gene family were proposed before the whole complement of FGF genes was described in mammals which led to incomplete conclusions [5, 8]. The first phylogenetic studies including all the mammalian FGFs proposed a division of the gene family into six [9] or seven [6] subfamilies. In 2005, Popovici and collaborators performed the first study including both protostome and deuterostome FGFs as well as FGFs from baculoviruses, an arthropod-specific group of viruses [4]. They proposed to divide the FGF gene family into eight subfamilies: subfamily A (including orthologs of FGF 1 and 2), subfamily B (orthologs of FGF 3, 7, 10, and 22), subfamily C (orthologs of FGF 4, 5, and 6), subfamily D (orthologs of FGF 8, 17, 18, and 24 from vertebrates but also of EGL-17, PYR, and THS from protostomes), subfamily E (orthologs of FGF 9, 16, and 20 but also of LET-756 from nematodes), subfamily F (orthologs of FGF 11, 12, 13, and 14), subfamily G (orthologs of FGF 15/19, 21, and 23), and subfamily H which is specific of arthropod FGFs (i.e., BNL) and of FGFs found in arthropod-specific viruses [4]. This classification is widely accepted today, however, the phylogenetic position of FGF3 and FGF5 is not completely solved, which calls into question the constitution of the two subfamilies B and C. Moreover, the description of FGF genes in the sea anemone *Nematostella vectensis* now raises the question of the timing of the appearance and diversification of the FGF gene family.

In this study we take advantage of the exponential increase of publicly available genomic sequences to present an update of the FGF gene content in different evolutionary lineages. Phylogenetic approaches, together with synteny conservation analyses of these data, allow us to propose a new classification of the FGF gene family which (i) confirms the paralogy relationships of the FGF4/5/6 subfamily members and (ii) suggest that orthologs of the mammalian FGF3 form a new subfamily.

## 2. The FGF Gene Content Varies among ****Different Metazoan Lineages

The recent development of high throughput sequencing techniques has generated a large number of sequences available in different public databases. Among them we have searched for FGF domain coding sequences within the major metazoan phyla, in order to clarify the evolutionary history of this family. We have limited our study to the analysis of amino acid sequences deposited in the Genbank, the Ensembl, and the JGI databases for cnidarians, lophotrochozoans, ecdysozoans, and deuterostomes, although many ESTs sequences putatively coding for FGF proteins might also be found. 

### 2.1. FGF Genes in Diploblastic Metazoans

FGF genes were previously described in two anthozoan species: *Nematostella vectensis* and *Acropora millepora* [10, 11]. In *Nematostella*, 13 genes encoding FGF ligands were predicted from the genome sequence [11] but their phylogenetic relationships with bilaterian FGFs are not fully established. Four of these genes group with the FGF8/17/18/24 subfamily and six group with the FGF1/2 subfamily with low support. In the hydrozoan *Hydra magnipapillata* we have found 4 predicted genes coding for FGFs (see Table 1). Among them, one (called FGF24) belongs to the FGF8/17/18/24 subfamily. Another one groups with several *Nematostella* FGF genes whose position is not robustly supported but might belong to the FGF1/2 subfamily (see Figure S1 in supplementary material available online at doi:10.1155/2012/298147). For the other two, no clear relationship with either *Nematostella* or bilaterian FGFs can be proposed according to phylogenetic reconstructions. We also looked for ctenophore EST sequences putatively encoding FGF domains but we failed to find any in public databases.

### 2.2. FGF Genes in Protostomes

In protostomes, FGF genes have only been described in ecdysozoans, particularly in arthropods. Three genes have been characterized in the model organism *Drosophila melanogaster* [12, 13], called *Branchless* (*Bnl*), *Thisbe* (*Ths*), and *Pyramus* (*Pyr*). In the coleopteran *Tribolium castaneum*, four FGF genes called *Tc-FGF1a, Tc-FGF1b, Tc-FGF8,* and *Tc-Bnl* [14] have also been identified. *Ths* and *Pyr* from *Drosophila*, as well as *Tc-FGF8* from *Tribolium*, were shown to belong to the FGF8/17/18/24 subfamily, whereas *Tc-FGF1a*, and *TcFGF1b* belong to the FGF1/2 subfamily. On the other hand, *Branchless* orthologs from both species show no clear evolutionary relationships with any of the vertebrates FGF gene subfamilies leading Popovici and collaborators to propose a new subfamily including *Bnl* from arthropods and baculovirus-specific FGF genes [4]. In the genome of the nematode *Caenorhabditis elegans* two FGF genes are found called *let-756* (lethal protein 756) and *egl-17* (egg laying defective 17) [4, 15], which are members of the FGF9/16/20 and FGF8/17/18/24 subfamilies, respectively [4].

In order to obtain a more complete picture of the diversity of the FGF gene family in ecdysozoans, we searched other available sequences (see Table 1). Thus, in different nematode species we only found orthologs of the two known *C. elegans* genes (Figure S2). In arthropods, we found FGF coding genes in the crustacean *Daphnia pulex*, in the chelicerate *Ixodes scapularis*, and in insects from different classes such as *Apis mellifera*, *Harpegnathos saltator,* or *Pediculus humanus* (see Table 1). The orthology relationships of the two FGF genes we found in *Daphnia* cannot be clearly determined, whereas for all the other arthropods the different genes we found always belong to the *Bnl*, FGF1/2, or FGF8/17/18/24 subfamilies (Figure S2).

No study of the FGF gene set in lophotrochozoans has been published yet so we searched for lophotrochozoan FGF coding sequences in Genbank and in the complete genome sequences of the mollusc *Lottia gigantea* and of the annelids *Helobdella robusta* and *Capitella teleta*. We found only one gene in *Capitella* whose position in the FGF phylogenetic tree is not robustly supported, but probably belongs to the FGF8/17/18/24 subfamily. In *Lottia gigantea*, two FGF genes are present in the complete genome, and again their evolutionary relationship with the different subfamilies cannot be clearly determined even if the best blast hit results for these genes are always orthologs of the FGF8/17/18/24 and FGF9/16/20 subfamilies (see Table 1). Taken together, these data demonstrate (i) that lophotrochozoans also possess some FGF coding genes, although quite divergent from the other protostome genes, and (ii) that members of only four subfamilies, FGF1/2, FGF8/17/18/24, FGF9/16/20, and *Bnl*, can be clearly found in protostomes.

### 2.3. FGF Genes in Deuterostomes

Deuterostomes comprise vertebrates, the related invertebrate chordates (urochordates and cephalochordates) and three other invertebrate taxa: hemichordates and echinoderms, which form the Ambulacraria group, and the recently described phylum of Xenoturbellida [16]. Nothing is known concerning the FGF gene content in Xenoturbella and we did not find any FGF coding sequence for this group. Conversely, recent studies have shown that one FGF gene exists in the sea urchin *Strongylocentrotus purpuratus* (i.e., echinoderm) [17], and we have identified in the databases six FGF genes in the hemichordate *Saccoglossus kowalevskii* of which one gene can be clearly assigned to the FGF8/17/18/24 subfamily. Three other genes are orthologs of the FGF9/16/20 subfamily, indicating that an hemichordate-specific duplication occurred for this gene; another one has been previously shown to be ortholog of the FGF19/21/23 [18]; the sixth gene shows no clear orthology relationships with any FGF gene subfamily (see Table 1) [18].

In chordates, the FGF gene content is also different among the three subphyla. In cephalochordates, eight FGF genes have been found and orthology relationships using phylogenetics or conservation of synteny approaches have been suggested for six of them (i.e., FGF1/2, FGF8/17/18, FGF9/16/20, FGFA ortholog of FGF3/7/10/22, FGFB ortholog of FGF4/5/6, and FGFC ortholog of FGF19/21/23) [19]. In the urochordate *Ciona intestinalis*, six genes encoding FGF ligands have been described [20], and we identified one more gene in databases, called FGF-NA1, bringing the total FGF gene content to seven. Of them, only two were shown to be clear orthologs of the FGF8/17/18/24 and FGF11/12/13/14 subfamilies [20]. In another urochordate, the larvacean *Oikopleura dioica*, we found six FGF coding genes, among which two can be assigned to the FGF11/12/13/14 subfamily, and one to the FGF9/16/20 subfamily (see Table 1 and Figure S4). In vertebrates, an explosion in the number of genes encoding FGFs occurred and we can find between 19 and 27 FGF genes depending on the species. This explosion is not specific to the FGF gene family and is linked to the two rounds of genome duplication (three rounds in teleosts) that occurred in this lineage as previously demonstrated [4, 21]. In sarcopterygians we identified 19 FGF genes in the chicken and 23 in the coelacanth, whereas 22 FGF genes (FGF 1–23) have been characterized in mouse and human (the mouse FGF15 is the ortholog of the human FGF19). These 22 mammalian genes were previously used to reconstruct the evolutionary history of the family [4, 6], which led to the classification of FGFs into seven paralogy groups. However, in teleosts, an additional round of genome duplication (3R hypothesis) occurred [22], which, together with a high number of FGF gene losses, produced 27 FGF genes in the zebrafish [23].

## 3. The FGF Gene Family Is Composed by Eight Subfamilies

**Figure 1:**
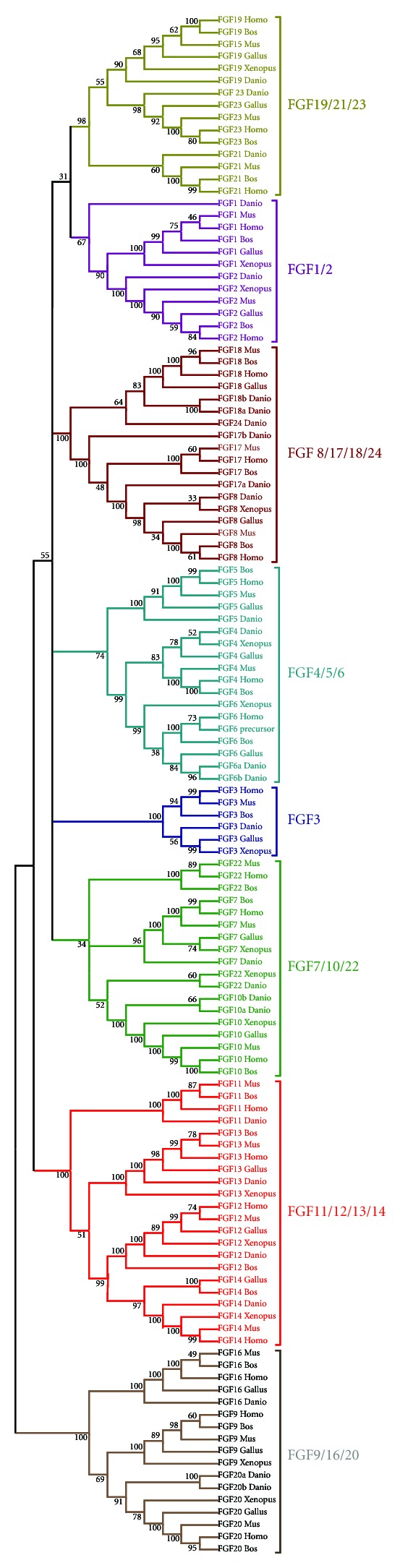
FGF phylogeny in vertebrates. Neighbor-joining tree showing the classification into eight subfamilies of the different vertebrate FGF genes (i.e., FGF1/2, FGF3, FGF4/5/6, FGF7/10/22, FGF8/17/18/24, FGF9/16/20, FGF11/12/13/14, and FGF19/21/23). Sequences of *Homo sapiens*, *Mus musculus*, *Bos taurus*, *Gallus gallus*, *Xenopus tropicalis*, and *Danio rerio* were used to perform the phylogeny.

Due to the low sequence conservation of most of the FGF genes found in early divergent metazoan lineages, and the short length of the FGF domain, we have based our phylogenetic study on vertebrate FGFs, as in previous studies [4, 6]. However, the new FGF sequence data, particularly within chordates, allow us to suggest a new classification of the FGF gene family in metazoans, which is divided into 8 subfamilies instead of 7 (in addition to the arthropod + baculoviruses—specific family proposed by Popovici et al. [4]). These families are the FGF1/2, FGF3, FGF4/5/6, FGF7/10/22, FGF8/17/18/24, FGF9/16/20, FGF11/12/13/14 and FGF19/21/23 (Figures 1 and S5).

**Figure 2:**
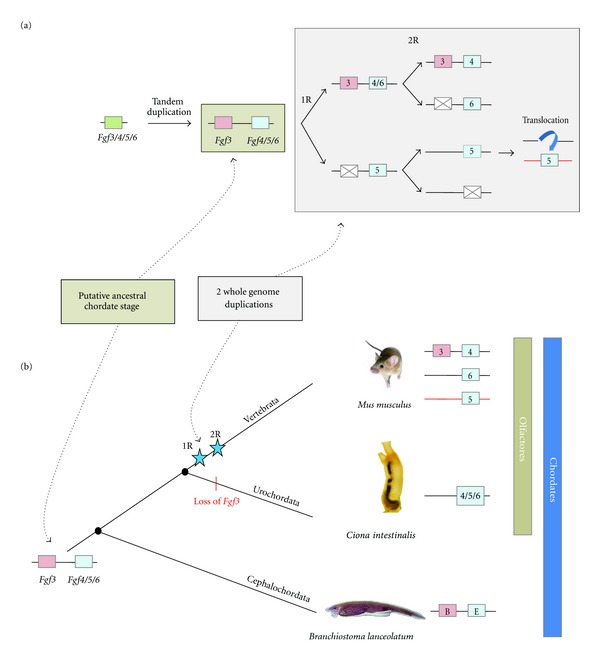
Evolutionary scenario of the FGF3 subfamily. (a) Genomic events leading to the birth of the FGF3 subfamily. From a single *FGF3/4/5/6* gene, a tandem duplication occurred before the chordate diversification giving rise to an *FGF3* and an *FGF4/5/6* gene (brown box). The two rounds of whole genome duplication, followed by several gene losses and by a specific translocation of the chromosome region containing *FGF5* (grey box) conducted to the gene content currently found in vertebrates. (b) Evolutionary relationships between FGFs 3, 4, 5, and 6 in chordates. Here, the chordate ancestor had both *FGF3* and *FGF4/5/6*. This gene content was kept in amphioxus, whereas *FGF3* was lost in urochordates and different gene losses account in vertebrates for the presence of a single FGF3 gene and three genes of the FGF4/5/6 paralogy group. This implies that in amphioxus *FGF3* and *FGFB* are orthologs, as well as *FGF4/5/6* and *FGFE*.

In all the studies performed so far, the vertebrate FGF3 always grouped into either the subfamily FGF3/7/10/22 or the subfamily FGF3/4/6 [4, 6, 8]. In fact, the correct classification of FGF3 is still debated and assignment to one or another subfamily depends on the methods used. Therefore, most of the phylogenetic analyses published grouped FGF3 with FGF7, FGF10, and FGF22, but with very low node robustness. Other studies, using the genomic locations of this gene, grouped it with FGF4 and FGF6 and it has even been suggested that the FGF3/4/6 and FGF19/21/23 subfamilies can be assembled into a single subfamily FGF3/4/6/19/21/23 (with FGF5 grouping in this case with the FGF1/2 subfamily) [7]. Here, based particularly on results obtained through the study of gene content, phylogenetic distribution, and conservation of synteny between amphioxus and vertebrates [19], we propose a new evolutionary scenario in which FGF3 forms a new subfamily (Figures 1, 2, and S5). This scenario could reconcile the different evolutionary hypotheses suggested in previous studies.

**Figure 3:**
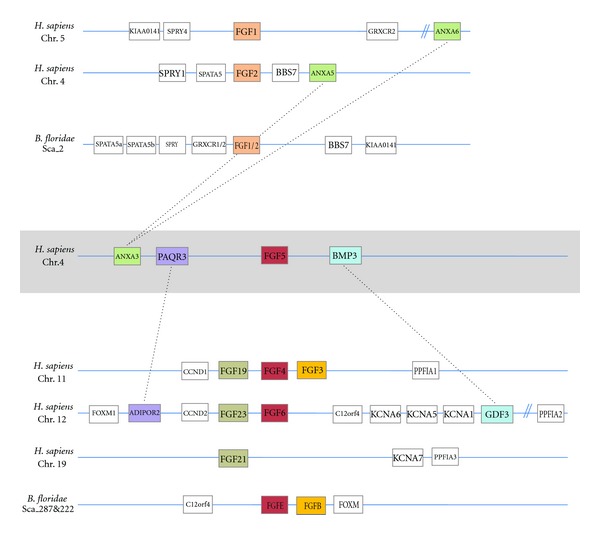
Chromosomal maps of human and amphioxus FGF1/2 and FGF4/5/6 genes loci. Synteny is well conserved among vertebrates and amphioxus for FGF1/2 (orange—upper part) and for FGF4/6 (red), which are also syntenic with FGFs 19/21/23 (brown) and with FGF3 (yellow—lower part). The synteny of FGF5 with BMP3, PAQR3, and ANXA3 suggests that this gene belongs to the FGF4/5/6 subfamily, but was probably secondarily translocated with his neighboring genes (BMP3, PAQR3, etc.) close to ANXA3.

In our hypothesis, an ancestral FGF gene (named FGF3/4/5/6) was duplicated in tandem before chordate diversification. Such duplication might have occurred before eumetazoan diversification or specifically in the chordate ancestor. Thus, the putative ancestor (either eumetazoan or chordate ancestor) had two FGF genes maintained in cluster: FGF3 and FGF4/5/6. This situation can still be observed in the cephalochordate *Branchiostoma floridae *in which FGFB and FGFE are clustered in a genomic region showing synteny conservation with the vertebrate locus containing the FGFs 3, 4 and 6 [19] (Figure 3). This hypothesis implies a loss of FGF3 in different lineages, the number of lineages that lost FGF3 depends on the timepoint at which this gene appeared (i.e., in urochordates in one hypothesis (Figures 2(b) and 5), or in urochordates, ambulacrarians, protostomes, and cnidarians in the other hypothesis, see Figure 5). According to this scenario the origin of FGF3 would be ancient (i.e., at least prior to chordates diversification) and not due to the vertebrate-specific genome duplications.

Another FGF gene whose phylogenetic position is debated is FGF5. Indeed, depending on the phylogenetic approach and on the gene set used for the phylogenetic reconstruction, it clusters either with FGF4/6 or with FGF1/2 [4, 23]. Moreover, conservation of synteny also suggests the paralogy of FGF1, 2, and 5 [7]. However, a deeper synteny analysis of the human FGF5 locus shows conservation of this locus with both the FGF1/2 and FGF4/6 loci (Figure 3). This mixed syntenic conservation, together with our phylogenetic analyses supporting the FGF4/5/6 subfamily (Figure 1), suggests that FGF5 is a real paralog of FGF4 and 6. The partial synteny conservation with the FGF1 and 2 loci might be explained by a genomic translocation of the FGF5 locus (including its neighbouring genes BMP3, PAQR3) close to the ANXA3 locus (Figures 2(a) and 3).

## 4. The Evolutionary History of the FGF Gene Family Is Characterized by Gene Duplications and Gene Losses

**Figure 4:**
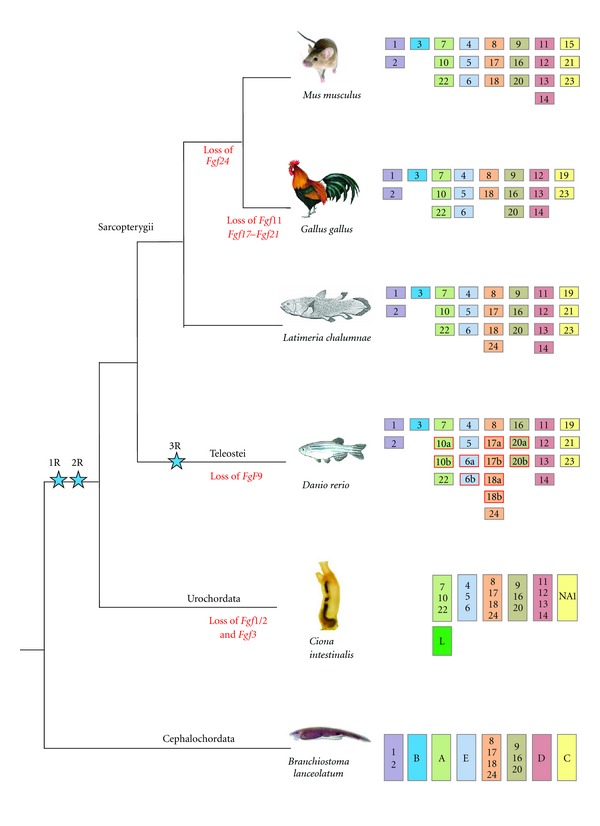
FGF gene content in chordates. Each of the eight FGF paralogy groups is represented by one color. Gene losses are indicated under the tree branches and specific teleost duplications are outlined in red. The urochordate FGFL which is considered as a specific duplication of FGF7/10/22 in this group is colored in dark green. Blue stars represent genome duplications.

**Figure 5:**
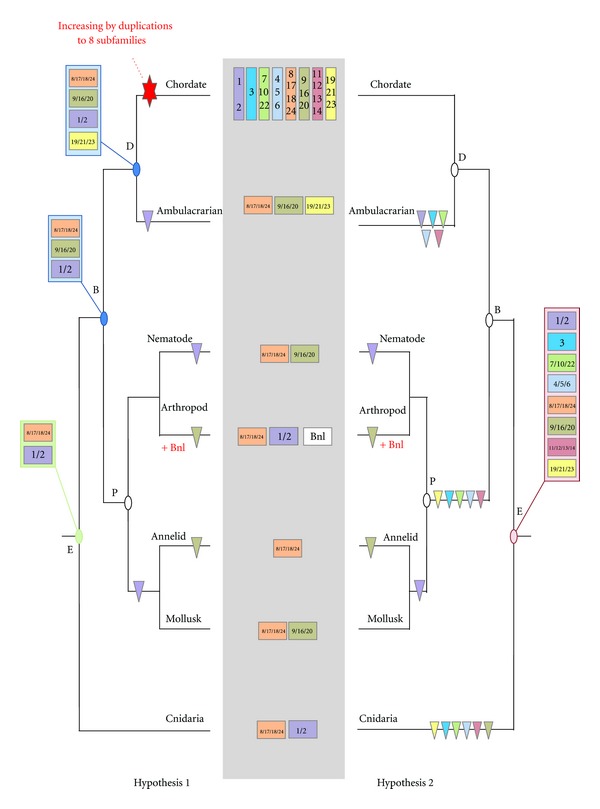
Evolutionary scenarios for FGF evolution in eumetazoans. The minimal gene content of each eumetazoan lineage (chordates, ambulacrarians, nematodes, arthropods, annelids, mollusks, and cnidarians) is mentioned in the center (grey box). Two evolutionary hypotheses are proposed: on the left side (hypothesis 1), starting from a minimum gene set of two genes (green box) in the eumetazoan ancestor, diversity of the subfamily is acquired through chordate-specific duplications; on the right side (hypothesis 2), diversity of the subfamily was acquired very early in metazoan evolution, with 8 subfamilies in the eumetazoan ancestor (red box) and then numerous gene losses in the different lineages occurred. Gene losses are represented by triangles. E: eumetazoan ancestor; P: protostome ancestor; D: deuterostome ancestor and B: Bilaterian ancestor.

Phylogenetic reconstructions using FGF sequences from all metazoan phyla often fail to completely solve the orthology relationship between the different members of this family mainly because of the reduced size of the FGF domain and because of the high divergence of the sequences between the different lineages. However, using the phylogenetic distribution of FGF genes into eight subfamilies, we can propose evolutionary scenarios accounting for the FGF gene content found in the different metazoan lineages. Several hypotheses can be drawn explaining such a distribution of FGF orthologs. Here we focus mainly on two of these hypotheses: a first hypothesis where the eight FGF subfamilies are chordate-specific (Figures 4 and 5, hypothesis 1) and a second hypothesis where the eight subfamilies were ancestral to all eumetazoans (Figure 5, hypothesis 2). In both hypotheses, the evolutionary history of the FGF gene content in chordates is the same (Figure 4), but depending on the hypothesis, it changes for the other metazoan lineages (Figure 5).

As we have shown, in cnidarians (diploblastic metazoans) we found the presence of, at least, orthologs of the FGF8/17/18 and probably FGF1/2 subfamilies. Thus, we can suggest that the eumetazoan ancestor possessed at least one ortholog of these two subfamilies.

Our analyses suggest that the arthropod ancestor already possessed at least three FGF genes belonging to the FG1/2, FGF8/17/18 and *Bnl* subfamilies (Figure 5). *Bnl* is specific to arthropods and arthropod viruses and its origin is still unknown. Two possible evolutionary scenarios can be drawn for *Bnl* genes. In the first scenario, a *Bnl* ortholog might have existed ancestrally and then been lost in all metazoan lineages except arthropods. Then this gene was captured by baculoviruses after the arthropod radiation [4]. In a second scenario, an arthropod FGF gene was translocated into baculoviruses and, following a period of fast evolution leading to the loss of any phylogenetic signal, reintegrated into the arthropod genome. In the ancestor of nematodes, two FGF genes, orthologs of the FGF9/16/20 and FGF8/17/18/24 families were present. Taking these results into account, we can propose the existence of a minimal FGF gene set of three genes in the ancestor of ecdysozoans (orthologs of FGF1/2, FGF8/17/18/24 and FGF9/16/20). The few data obtained in lophotrochozoans do not allow us to clearly conclude on the FGF gene set of the protostome ancestor. However, we can suggest the presence of at least members of the FGF1/2, FGF8/17/18, and FGF9/16/20 subfamilies.

The two hypotheses proposed here for the evolutionary history of the FGF gene family (Figure 5) suggest that a single paralogous gene for each subfamily was kept in cephalochordates and that specific gene duplications or losses did not occur during evolution in this lineage (Figure 4). In fact, genetic conservation in amphioxus is not restricted to FGFs since different studies have shown that gene content in amphioxus tends to be associated with very few gene losses [24–28]. Concerning other chordates, even if the phylogenetic distribution of the seven urochordate FGF genes is not strongly supported (see Figure S4), we can assume that *C. intestinalis* has orthologs of the FGF4/5/6, FGF7/10/22, FGF8/17/18, FGF9/16/20, FGF11/12/13/14, and FGF19/21/23 subfamilies but that it lost the orthologs of the FGF1/2 and FGF3 subfamilies (Figure 4). Moreover, the seventh gene (Ci-FGFL), as proposed by Popovici et al., could be a specific duplication of FGF7/10/22 [4]. In sarcopterygian vertebrates, the gene set of the different species suggests that numerous gene losses occurred following the two rounds of genome duplication (from eight ancestral genes, after two rounds of duplication, we should find 32 genes, but depending on the species we find between 19 and 23 genes—Figure 4). Moreover, some lineage-specific gene losses also occurred in sarcopterygians; for example, the loss of FGF24 in tetrapods and losses of FGF11, 17, and 21 in chicken. In teleosts, gene losses were even more important, since instead of 46 genes (i.e., a duplication of the 23 FGF genes present in the osteichthyan ancestor [22]) we only find 27 in zebrafish [23]. Indeed, duplicated copies generated by this third genome duplication were only retained for FGF10, FGF6, FGF17, FGF18, and FGF20 (Figure 4).

In non-chordate deuterostomes, the only FGF gene found in the sea urchin cannot be assigned to any FGF subfamily using phylogenetic reconstructions, whereas five of the six genes found in *S. kowalevskii* belong to the FGF8/17/18/24, FGF9/16/20, and FGF19/21/23 subfamilies (Figure S3) [18]. The remaining gene does not show clear phylogenetic relationships with the different FGF subfamilies. Therefore, whatever the evolutionary hypothesis (i.e., chordate-specific duplications versus early duplication giving rise to eight subfamilies in the ancestral eumetazoan), we can propose that there were at least three FGF genes in the ambulacrarian ancestor (i.e., orthologs of FGF8/17/18/24, FGF9/16/20, and FGF19/21/23) (Figure 5). This result suggests that the deuterostome ancestor had probably at least these three genes plus FGF1/2 which is present in chordates and in protostomes but seems to be lost in the Ambulacraria. At this stage of the analysis it is difficult to say if specific chordate duplications led to the eight chordate FGFs (hypothesis 1, Figure 5), or if there was already eight genes in the deuterostome ancestor, several of them having being lost in Ambulacraria (hypothesis 2, Figure 5).

Here, for simplicity, we showed two extreme scenarios, one starting from the minimum gene set in the eumetazoan ancestor (only two genes) and the second starting from the maximum (eight genes). However, many other intermediate scenarios can be imagined. These two major evolutionary scenarios (Figure 5) imply different duplication/loss evolutionary histories. The first hypothesis implies two main points: (i) the ancestral eumetazoan had an FGF gene set of at least two genes (orthologs of FGF1/2 and FGF8/17/18/24) and (ii) important chordate-specific duplications occurred generating the present diversity of the FGF gene family observed in this lineage, which is divided into eight subfamilies (hypothesis 1, Figure 5). The second scenario implies a high degree of gene losses during metazoan evolution. Thus, from eight ancestral FGF gene families already present in the eumetazoan ancestor, six gene losses occurred in cnidarians, five in protostomes and five in ambulacrarians (hypothesis 2, Figure 5). Moreover, both hypotheses require lineage-specific duplications. The second hypothesis is less parsimonious than the first, but no matter which is correct, what seems clear is that the evolutionary history of the FGF gene family required numerous events of gene duplication and gene loss at different times and in different evolutionary lineages. The next question we should address in the near future is which are the implications of this complicated evolutionary history of the FGF gene family on the functional evolution of this signal and in the morphological evolution of metazoans.

## 5. Materials and Methods

### 5.1. Identification of FGF Sequences

FGF sequences were identified using BLASTP search in the NCBI and JGI [25] databases using all known FGF domain amino acid sequences. We also browsed the Pfam database [29] for entries possessing an FGF domain. Sequence accession numbers of FGF sequences identified in this study are shown in Table 1.

### 5.2. Phylogenetic Analyses of Vertebrate FGFs

FGF amino acid sequences were aligned using clustalX [30] and regions of ambiguous homology were removed. Neighbour-Joining tree was generated using *MEGA* version 5 [31] with a Poisson model and a discrete gamma-distribution model with four rate categories. Maximum Likelihood (ML) tree was built using PHYML3.0 [32] with a JTT model as proposed by ProtTest2.4 [33]. The node robustness of both trees was estimated by a bootstrap test (100 replicates).

### 5.3. Phylogenetic Analyses of Nonvertebrate FGFs

The FGF domain coding region of retrieved sequences was aligned with known FGF sequences from metazoans using T-Coffee [34]. The resulting alignment was manually corrected in SeaView [32]. Maximum Likelihood (ML) trees were generated using PHYML3.0 [32] with a LG+G model as proposed by ProtTest2.4 [33]. The robustness of the tree nodes was estimated using aLRT.

## Supplementary Material


Figure 1: Phylogenetic relationships of vertebrate and cnidarian FGF genes. FGF1/2 and FGF8/17/18/24 families are yellow boxed. The aLRT support for the nodes of these families is encircled in red.
Figure 2: Phylogenetic relationships of vertebrate and protostome FGF genes. FGF1/2, FGF8/17/18/24 and FGF9/16/20 families are yellow boxed. The aLRT support for the nodes of these families is encircled in red.
Figure 3: Phylogenetic relationships of vertebrate and hemichordate FGF genes. FGF8/17/18/24 and FGF9/16/20 families are yellow boxed. The aLRT support for the nodes of these families is encircled in red.
Figure 4: Phylogenetic relationships of vertebrate and Oikopleura FGF genes. FGF11/12/13/14 and FGF9/16/20 families are yellow boxed. The aLRT support for the nodes of these families is encircled in red.
Figure 5: Phylogenetic relationships of vertebrate FGFs. Maximum likelihood tree showing the classification into eight subfamilies of the different vertebrate FGF genes (i.e. FGF1/2, FGF3, FGF4/5/6, FGF7/10/22, FGF8/17/18/24, FGF9/16/20, FGF11/12/13/14 and FGF19/21/23). Sequences of Homo sapiens, Mus musculus, Bos taurus, Gallus gallus, Xenopus tropicalis, and Danio rerio were used to perform the phylogeny. Branches of the eight subfamilies are highly supported (at least 68 %) but internal branches within the different subfamilies do not always follow the evolution of species.

## Figures and Tables

**Table 1:** FGF domain containing protein sequences used in this study. For each species the accession number of all the proteins found are given, as well as orthology when well supported in phylogenetic reconstructions. Best blastP hit are given when no clear orthologu relationships was found.

Species	Accession number	Description	Database	Best blastP hit accession	Best blastP hit name	Orthology
*Hydra magnipapillata*	XP_002165496.1	Predicted: similar to fibroblast growth factor homologous factor 4	Genbank	NP_001180935.1	Fibroblast growth factor 12 (*Macaca mulatta*)	
	XP_002164870.1	Predicted: similar to fibroblast growth factor 24	Genbank			FGF8/17/18
	XP_002166704.1	Predicted: similar to fibroblast growth factor 1B, partial	Genbank			FGF1/2
	XP_002170051.1	Predicted: similar to Fibroblast growth factor 14 (*Hydra magnipapillata*)	Genbank	XP_001094679.1	Predicted: fibroblast growth factor 20 (*Macaca mulatta*)	

*Lottia gigantea*	fgenesh2_pg.C_sca_ 110000014		JGI	XP_002643284.1	EGL-17 (*Caenorhabditis briggsae*)	
	fgenesh2_pg.C_sca_ 16000265		JGI	XP_003455818.1	Predicted: fibroblast growth factor 20-like (*Oreochromis niloticus*)	

*Capitella teleta*	fgeneshl_pg.C_scaffold_ 1200000001		JGI	XP_002922927.1	Predicted: fibroblast growth factor 18-like (*Ailuropoda melanoleuca*)	

*Trichinella spiralis*	XP_003370033	Fibroblast growth factor 20	Genbank	NP_001098209.1	Fibroblast growth factor 20a (*Oryzias latipes*)	
	EFV50493.1	Fibroblast growth factor 18	Genbank			FGF8/17/18

*Brugia malayi*	XP_001894505.1	Fibroblast growth factor family protein	Genbank			FGF9/16/20
	XP_001899322.1	Fibroblast growth factor family protein	Genbank			FGF8/17/18

*Apis mellifera*	XP_623927.2	Predicted: hypothetical protein LOC551529	Genbank			FGF1/2
	XP_001120331.2	Predicted: hypothetical protein LOC724469	Genbank			BNL
	XP_003695580.1	Predicted: fibroblast growth factor 18-like	Genbank			FGF8/17/18

*Harpegnathos saltator*	EFN80858.1	Hypothetical protein EAI_11890	Genbank	XP_003399646.1	Predicted: hypothetical protein LOCI00646960 (*Bombus terrestris*)	
	EFN81752.1	Fibroblast growth factor 18	Genbank			FGF8/17/18
	EFN88402.1	Heparin-binding growth factor 1	Genbank			FGF1/2

*Pediculus humanus subsp. corporis*	EEB17861.1	Fibroblast growth factor, putative	Genbank	XP_003243356.1	Predicted: hypothetical protein LOC100572243 (*Acyrthosiphon pisum*)	
	EEB19433.1	Heparin-binding growth factor 1 precursor, putative	Genbank			FGF1/2
	EEB18362.1	Conserved hypothetical protein	Genbank	XP_002431100.1	Predicted: hypothetical protein LOC 100569010 (*Acyrthosiphon pisum*)	

*Ixodes scapu* *laris*	XP_002433492.1	Hypothetical protein IscW_ISCW015993	Genbank	XP_003203489.1	Predicted: glia-activating factor-like (*Meleagris gallopavo*)	
	XP_002400933.1	Heparin-binding growth factor, putative	Genbank			FGF1/2

*Daphnia pulex*	EFX75093.1	Hypothetical protein DAPPUDRAFT_ 108237	Genbank	XP_003243356.1	Predicted: hypothetical protein LOC 100572243 (*Acyrthosiphon pisum*)	
	EFX86332.1	Hypothetical protein DAPPUDKAFT_ 98099	Genbank	XP_001635198.1	Predicted protein (*Nematostella vectensis*)	

*Saccoglossus kowalevskii*	ADB22412.1	Fibroblast growth factor 8/17/18 protein	Genbank			FGF8/17/18
	ADB22409.1	Hypothetical protein	Genbank	XP_799351.2		
	ACY92516.1	Fgf-Sk1protein	Genbank	NP_001233192.1		
	ACY92517.1	FGF9-like protein	Genbank			FGF9/16/20
	ACY92515.1	FGF13-like protein	Genbank			FGF9/16/20
	ADB22411.1	Fibroblast growth factor 20-like protein	Genbank			FGF9/16/20

*Oikopleura dioica*	CBY43668.1	Unnamed protein product	Genbank	XP_003441021.1	Predicted: fibroblast growth factor 14-like (*Oreochromis niloticus*)	
	CBY37156.1	Unnamed protein product	Genbank			FGF11/12/13/14
	CBY40156.1	Unnamed protein product	Genbank			FGF11/12/13/14
	CBY12333.1	Unnamed protein product	Genbank			FGF9/16/20
	CBY34733.1	Unnamed protein product	Genbank	NP_001007762.1	Keratinocyte growth factor precursor (*Danio rerio*)	
	CBY23701.1	Unnamed protein product	Genbank	XP_002594626.1	Hypothetical protein BRAFLDRAFT_149779 (*Branchiostoma floridae*)	

## References

[1] Trowell OA, Willmer EN (1939). Studies on the Growth of Tissues in vitro. *The Journal of Experimental Biology*.

[2] Gospodarowicz D, Jones KL, Sato G (1974). Purification of a growth factor for ovarian cells from bovine pituitary glands. *Proceedings of the National Academy of Sciences of the United States of America*.

[3] Kolpakova E, Wiedlocha A, Stenmark H, Klingenberg O, Falnes PO, Olsnes S (1998). Cloning of an intracellular protein that binds selectively to mitogenic acidic fibroblast growth factor. *Biochemical Journal*.

[4] Popovici C, Roubin R, Coulier F, Birnbaum D (2005). An evolutionary history of the FGF superfamily. *BioEssays*.

[5] Ornitz DM, Itoh N (2001). Fibroblast growth factors. *Genome Biology*.

[6] Itoh N, Ornitz DM (2004). Evolution of the Fgf and Fgfr gene families. *Trends in Genetics*.

[7] Itoh N (2007). The Fgf families in humans, mice, and zebrafish: their evolutional processes and roles in development, metabolism, and disease. *Biological and Pharmaceutical Bulletin*.

[8] Coulier F, Pontarotti P, Roubin R, Hartung H, Goldfarb M, Birnbaum D (1997). Of worms and men: an evolutionary perspective on the fibroblast growth factor (FGF) and FGF receptor families. *Journal of Molecular Evolution*.

[9] Kim HS (2001). The human FGF gene family: chromosome location and phylogenetic analysis. *Cytogenetics and Cell Genetics*.

[10] Technau U, Rudd S, Maxwell P (2005). Maintenance of ancestral complexity and non-metazoan genes in two basal cnidarians. *Trends in Genetics*.

[11] Matus DQ, Thomsen GH, Martindale MQ (2007). FGF signaling in gastrulation and neural development in Nematostella vectensis, an anthozoan cnidarian. *Development Genes and Evolution*.

[12] Sutherland D, Samakovlis C, Krasnow MA (1996). *branchless* encodes a Drosophila FGF homolog that controls tracheal cell migration and the pattern of branching. *Cell*.

[13] Stathopoulos A, Tam B, Ronshaugen M, Frasch M, Levine M (2004). Pyramus and thisbe: FGF genes that pattern the mesoderm of Drosophila embryos. *Genes and Development*.

[14] Beermann A, Schröder R (2008). Sites of Fgf signalling and perception during embryogenesis of the beetle *Tribolium castaneum*. *Development Genes and Evolution*.

[15] Burdine RD, Chen EB, Kwok SF, Stern MJ (1997). egl-17 encodes an invertebrate fibroblast growth factor family member required specifically for sex myoblast migration in *Caenorhabditis* elegans. *Proceedings of the National Academy of Sciences of the United States of America*.

[16] Bourlat SJ, Juliusdottir T, Lowe CJ (2006). Deuterostome phylogeny reveals monophyletic chordates and the new phylum Xenoturbellida. *Nature*.

[17] Lapraz F, Röttinger E, Duboc V (2006). RTK and TGF-*β* signaling pathways genes in the sea urchin genome. *Developmental Biology*.

[18] Pani AM, Mullarkey EE, Aronowicz J, Assimacopoulos S, Grove EA, Lowe CJ (2012). Ancient deuterostome origins of vertebrate brain signalling centres. *Nature*.

[19] Bertrand S, Camasses A, Somorjai I (2011). Amphioxus FGF signaling predicts the acquisition of vertebrate morphological traits. *Proceedings of the National Academy of Sciences of the United States of America*.

[20] Satou Y, Imai KS, Satoh N (2002). Fgf genes in the basal chordate Ciona intestinalis. *Development Genes and Evolution*.

[21] Dehal P, Boore JL (2005). Two rounds of whole genome duplication in the ancestral vertebrate. *PLoS Biology*.

[22] Jatllon O, Aury JM, Brunet F (2004). Genome duplication in the teleost fish Tetraodon nigroviridis reveals the early vertebrate proto-karyotype. *Nature*.

[23] Itoh N, Konishi M (2007). The zebrafish FgF family. *Zebrafish*.

[24] Takatori N, Butts T, Candiani S (2008). Comprehensive survey and classification of homeobox genes in the genome of amphioxus, *Branchiostoma* floridae. *Development Genes and Evolution*.

[25] Putnam NH, Butts T, Ferrier DEK (2008). The amphioxus genome and the evolution of the chordate karyotype. *Nature*.

[26] Huang S, Yuan S, Guo L (2008). Genomic analysis of the immune gene repertoire of amphioxus reveals extraordinary innate complexity and diversity. *Genome Research*.

[27] Holland LZ, Albalat R, Azumi K (2008). The amphioxus genome illuminates vertebrate origins and cephalochordate biology. *Genome Research*.

[28] D’Aniello S, Irimia M, Maeso I (2008). Gene expansion and retention leads to a diverse tyrosine kinase superfamily in amphioxus. *Molecular Biology and Evolution*.

[29] Punta M, Coggill PC, Eberhardt RY (2012). The Pfam protein families database. *Nucleic Acids Research*.

[30] Thompson JD, Gibson TJ, Plewniak F, Jeanmougin F, Higgins DG (1997). The CLUSTAL_X windows interface: flexible strategies for multiple sequence alignment aided by quality analysis tools. *Nucleic Acids Research*.

[31] Tamura K, Peterson D, Peterson N, Stecher G, Nei M, Kumar S (2011). MEGA5: molecular evolutionary genetics analysis using maximum likelihood, evolutionary distance, and maximum parsimony methods. *Molecular Biology and Evolution*.

[32] Gouy M, Guindon S, Gascuel O (2010). Sea view version 4: a multiplatform graphical user interface for sequence alignment and phylogenetic tree building. *Molecular Biology and Evolution*.

[33] Abascal F, Zardoya R, Posada D (2005). ProtTest: selection of best-fit models of protein evolution. *Bioinformatics*.

[34] Notredame C, Higgins DG, Heringa J (2000). T-coffee: a novel method for fast and accurate multiple sequence alignment. *Journal of Molecular Biology*.

