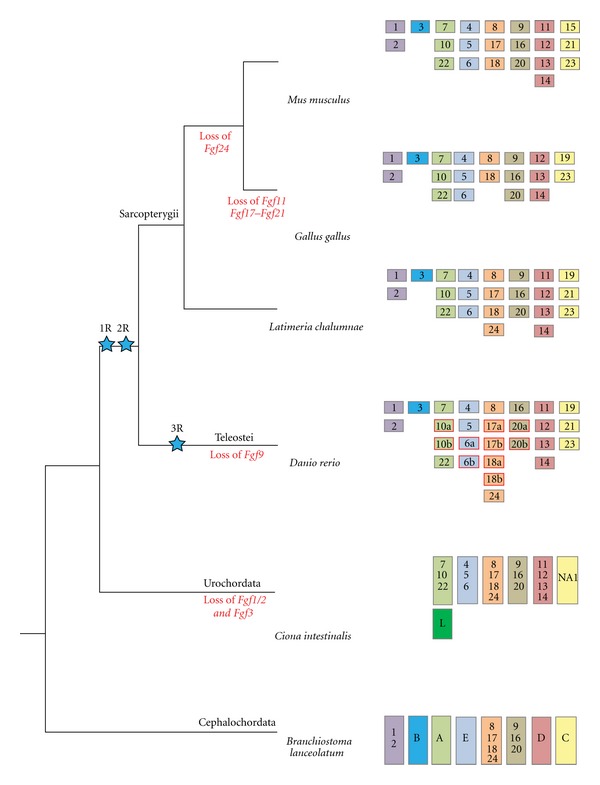# Erratum to “Evolution of the FGF Gene Family”

**DOI:** 10.1155/2012/789125

**Published:** 2012-11-11

**Authors:** Silvan Oulion, Stephanie Bertrand, Hector Escriva

**Affiliations:** CNRS, UMR 7232, BIOM, Université Pierre et Marie Curie Paris 06, Observatoire Océanologique, 66650 Banyuls-sur-Mer, France

The authors would like to make the following correction. In Figure  4 in the original paper shows two blue stars indicating genome duplications in the wrong place of the phylogenetic tree. This figure should be replaced by the following figure. The previous legend of Figure  4 is correct. 

## Figures and Tables

**Figure 4:**