# Supplementary figures and images for: Evolution of the FGF Gene Family
Source: Int J Evol Biol. 2012 Aug 7;2012:298147. doi: 10.1155/2012/298147 (PMC3420111; doi:10.1155/2012/298147)

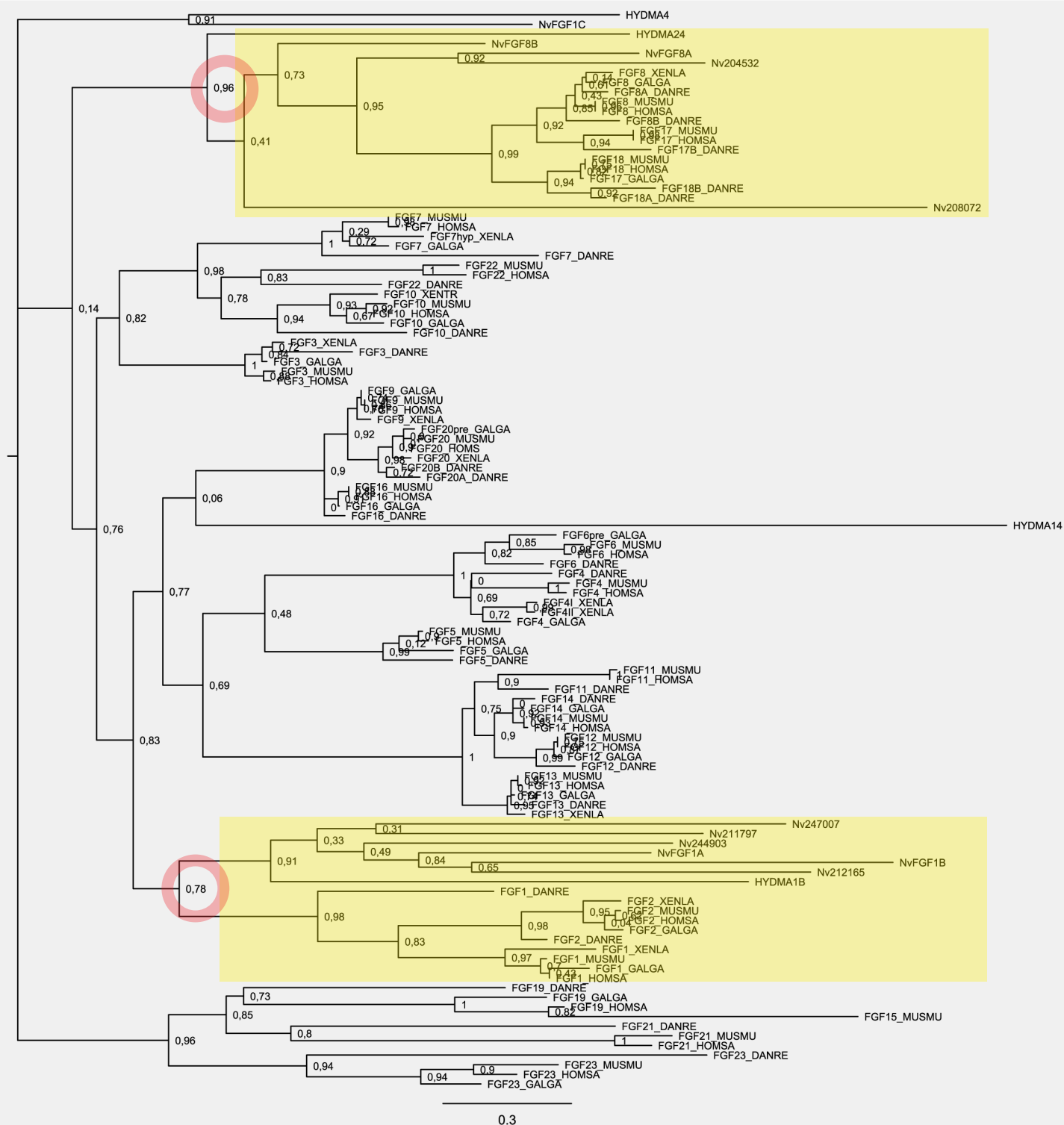

Supplement: Supplementary file 1 — Figure 1: Phylogenetic relationships of vertebrate and cnidarian FGF genes. FGF1/2 and FGF8/17/18/24 families are yellow boxed. The aLRT support for the nodes of these families is encircled in red. Figure 2: Phylogenetic relationships of vertebrate and protostome FGF genes. FGF1/2, FGF8/17/18/24 and FGF9/16/20 families are yellow boxed. The aLRT support for the nodes of these families is encircled in red. Figure 3: Phylogenetic relationships of vertebrate and hemichordate FGF genes. FGF8/17/18/24 and FGF9/16/20 families are yellow boxed. The aLRT support for the nodes of these families is encircled in red. Figure 4: Phylogenetic relationships of vertebrate and Oikopleura FGF genes. FGF11/12/13/14 and FGF9/16/20 families are yellow boxed. The aLRT support for the nodes of these families is encircled in red. Figure 5: Phylogenetic relationships of vertebrate FGFs. Maximum likelihood tree showing the classification into eight subfamilies of the different vertebrate FGF genes (i.e. FGF1/2, FGF3, FGF4/5/6, FGF7/10/22, FGF8/17/18/24, FGF9/16/20, FGF11/12/13/14 and FGF19/21/23). Sequences of Homo sapiens, Mus musculus, Bos taurus, Gallus gallus, Xenopus tropicalis, and Danio rerio were used to perform the phylogeny. Branches of the eight subfamilies are highly supported (at least 68 %) but internal branches within the different subfamilies do not always follow the evolution of species. [file 298147.f1.pdf]

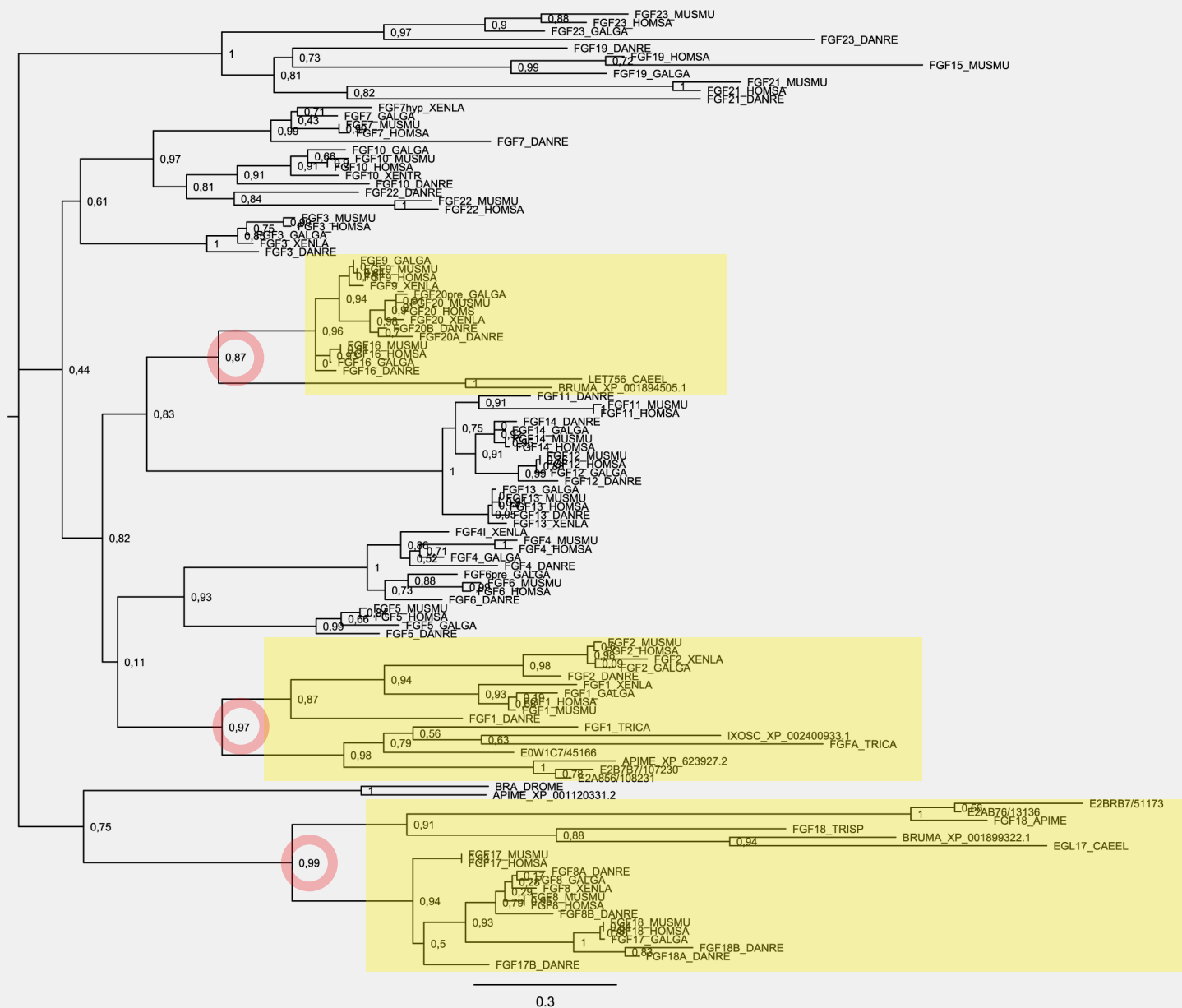

Supplement: Supplementary file 2 [file 298147.f2.pdf]

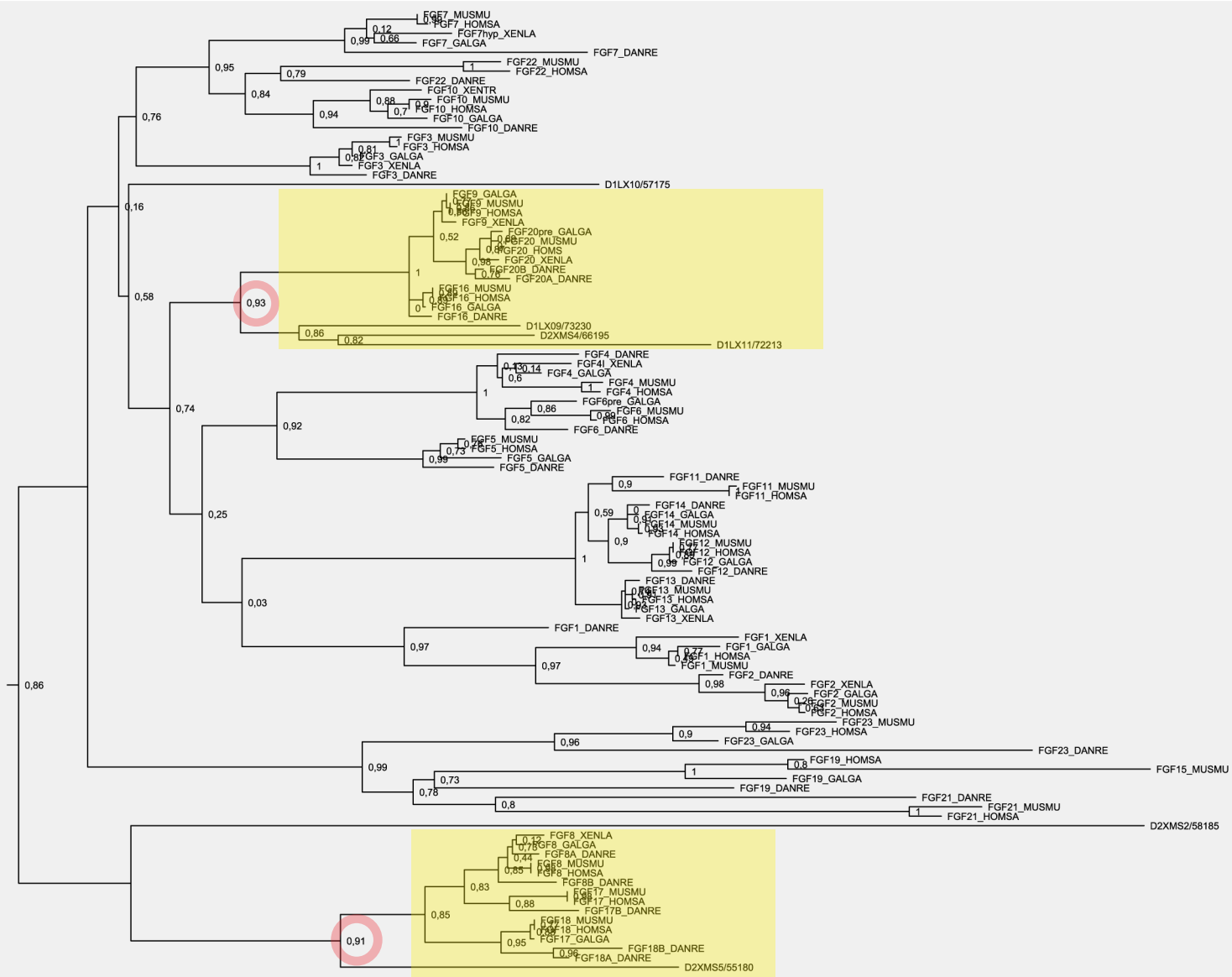

Supplement: Supplementary file 3 [file 298147.f3.pdf]

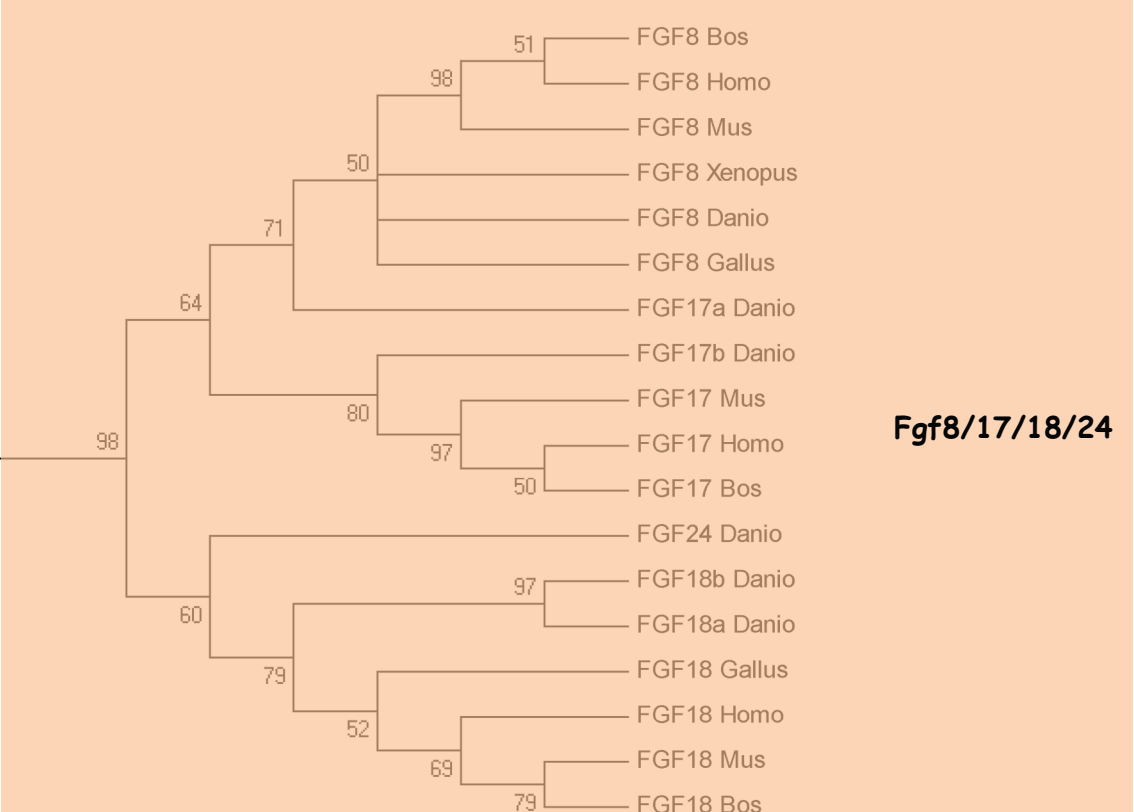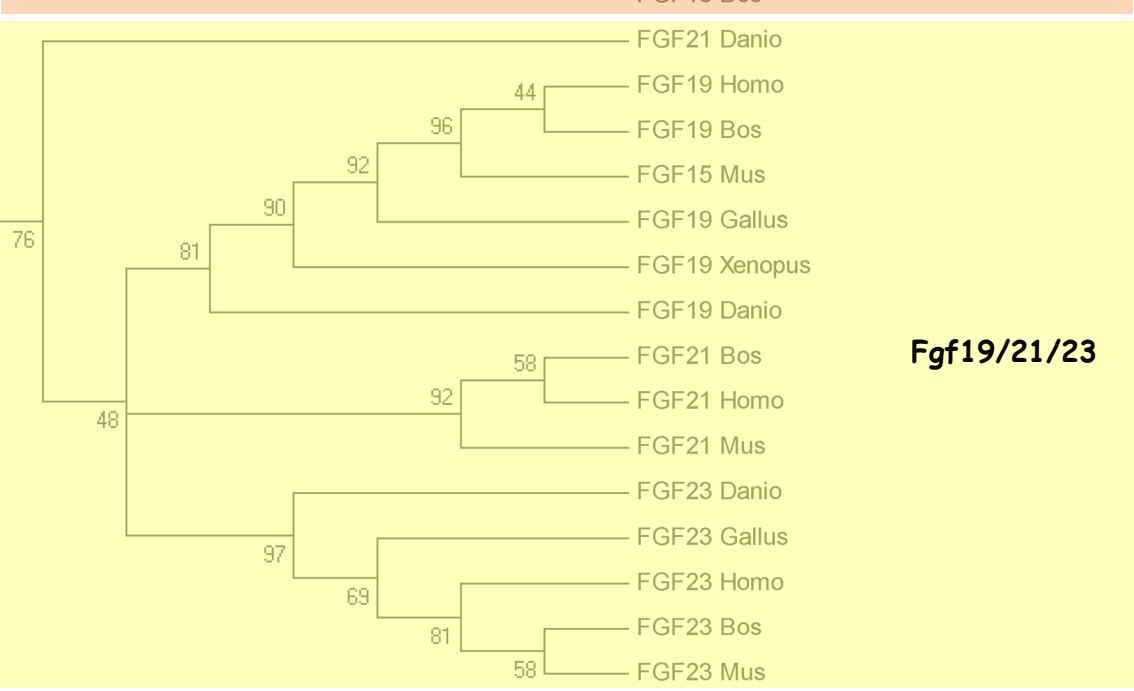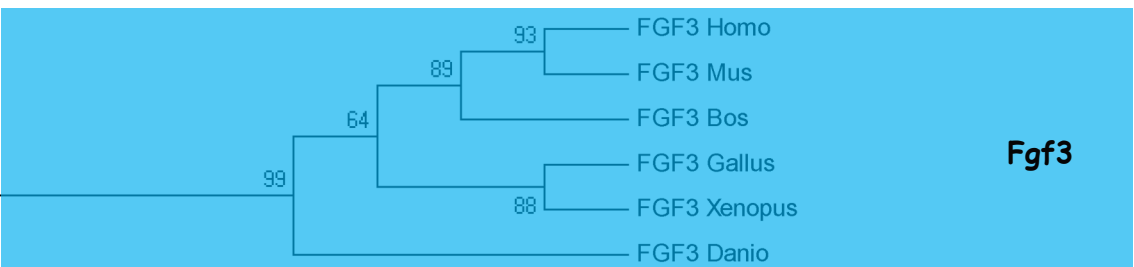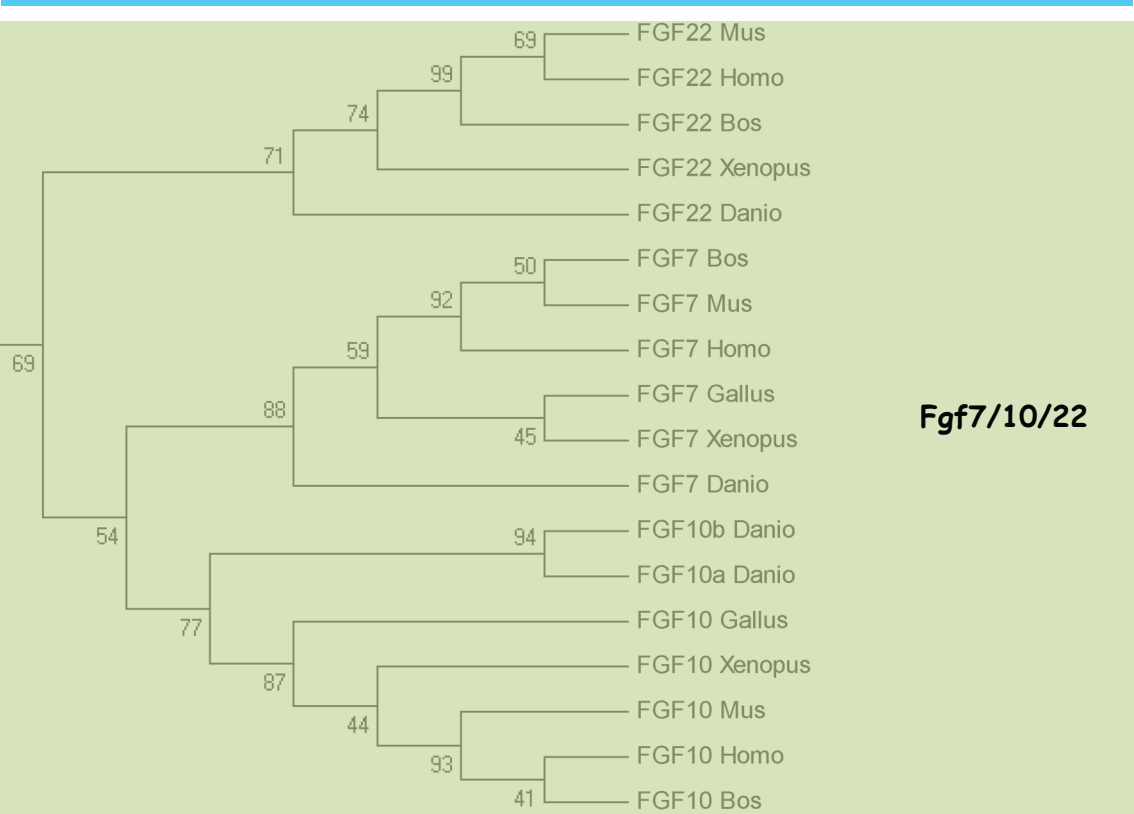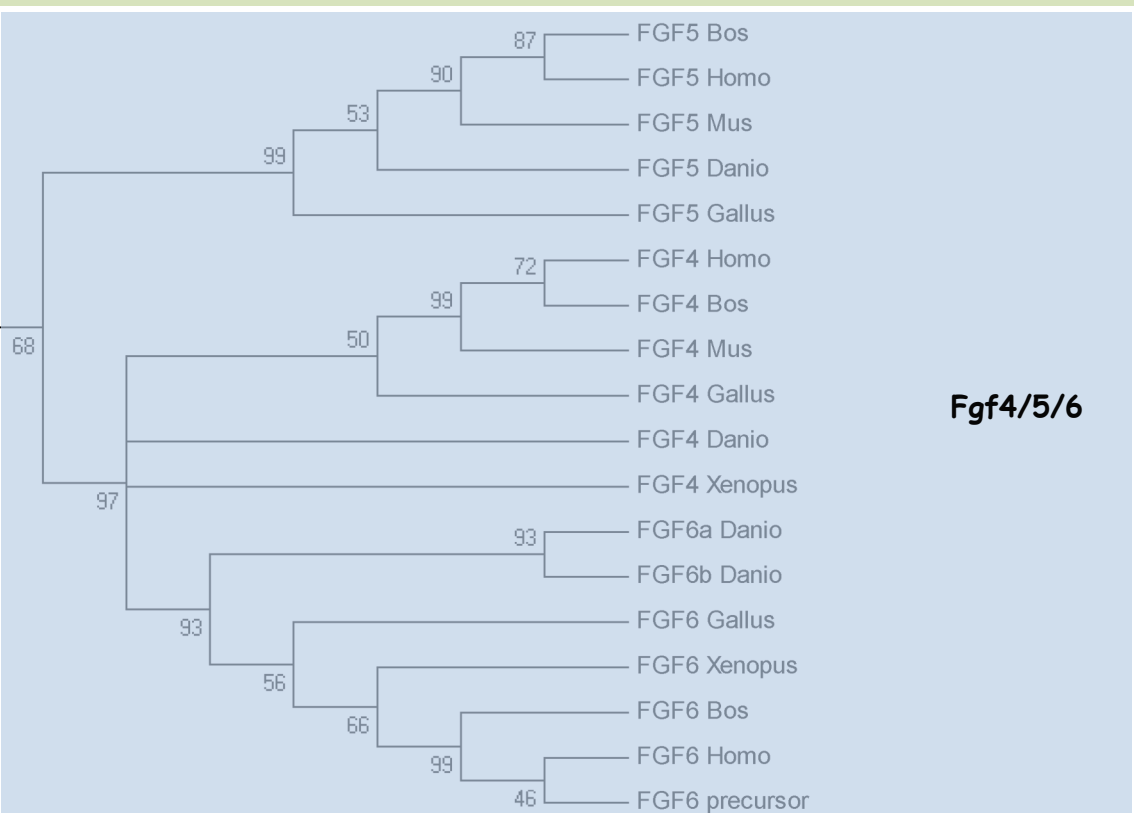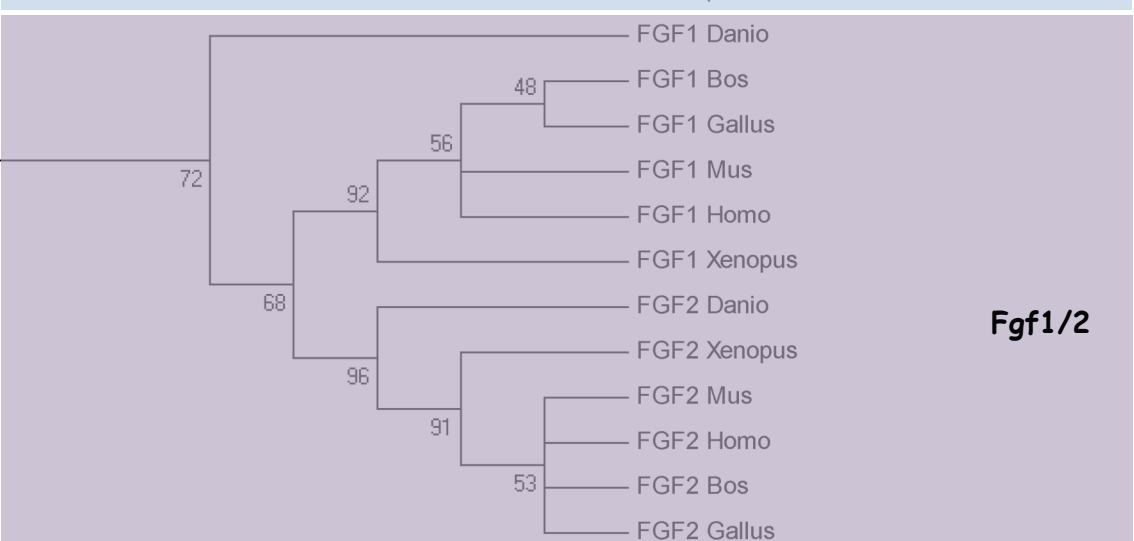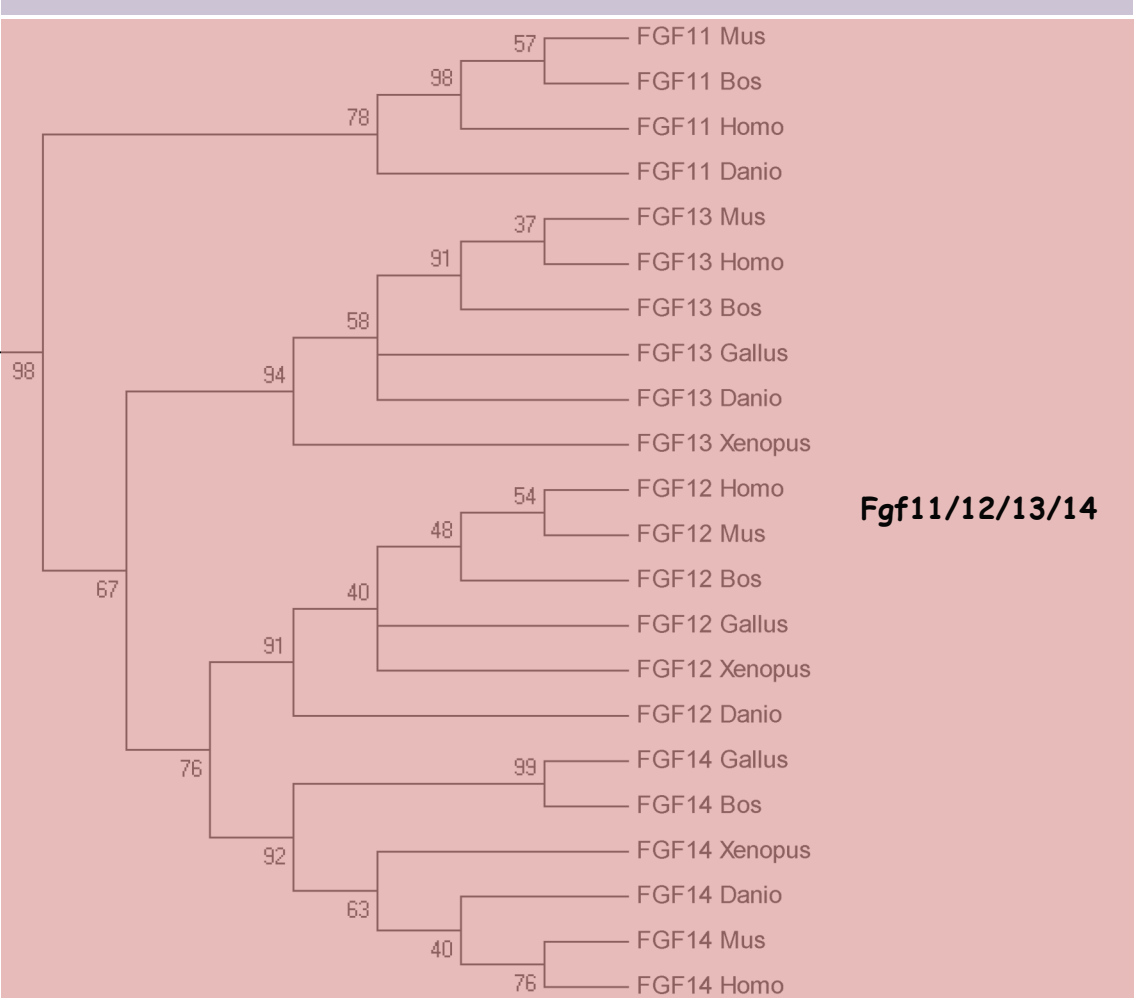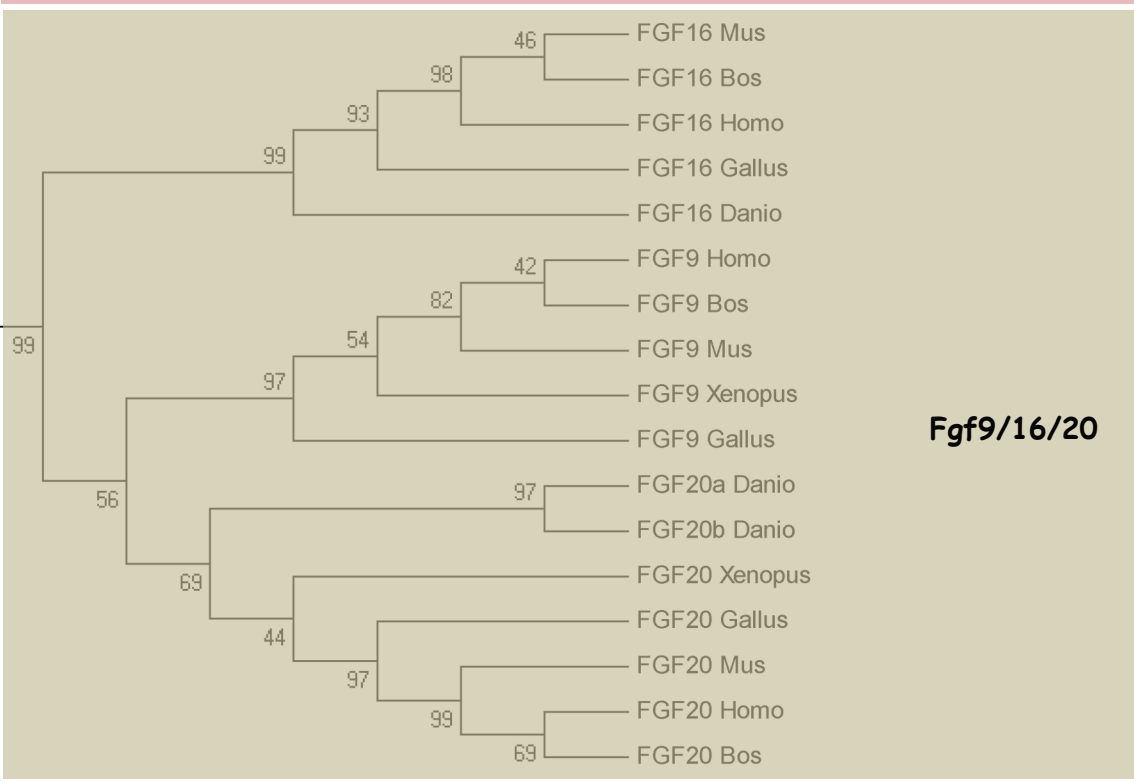

Supplement: Supplementary file 5 [file 298147.f5.pdf]
