# Supplementary material for: Evolution of the FGF Gene Family
Source: Int J Evol Biol. 2012 Aug 7;2012:298147. doi: 10.1155/2012/298147 (PMC3420111; doi:10.1155/2012/298147)
Supplement: Supplementary file 4 [file 298147.f4.pdf]

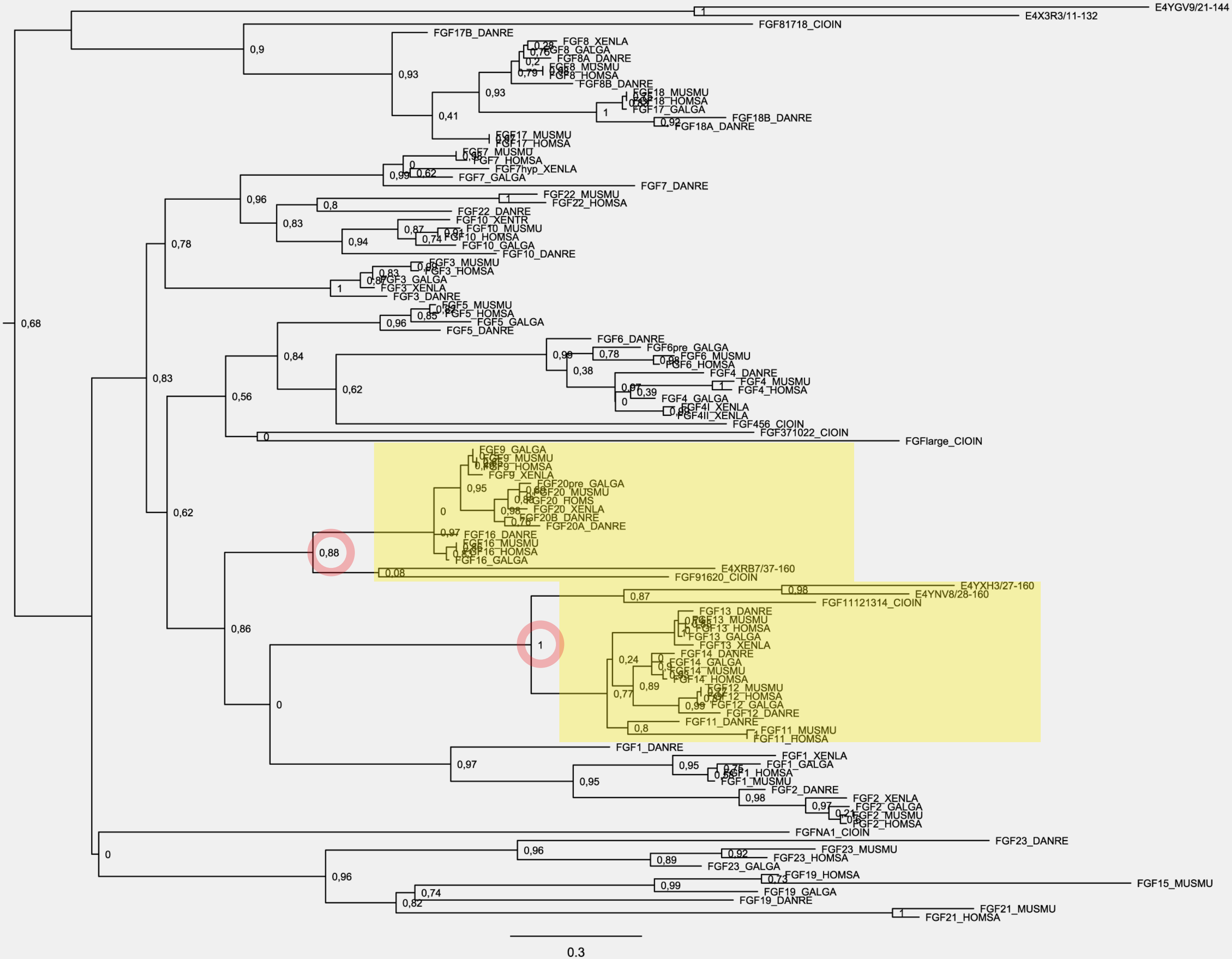

E4YGV9/21-144
E4X3R3/11-132
FGF81718_CIOIN
FGF17B_DANRE
FGF8_XENLA
FGF8_GALGA
FGF8A_DANRE
FGF8_MUSMU
FGF8_HOMSA
FGF8B_DANRE
FGF18_MUSMU
FGF18_HOMSA
FGF17_GALGA
FGF18B_DANRE
FGF18A_DANRE
FGF17_MUSMU
FGF17_HOMSA
FGF7_MUSMU
FGF7_HOMSA
FGF7hyp_XENLA
FGF7_GALGA
FGF7_DANRE
FGF22_MUSMU
FGF22_HOMSA
FGF22_DANRE
FGF10_XENTR
FGF10_MUSMU
FGF10_HOMSA
FGF10_GALGA
FGF10_DANRE
FGF3_MUSMU
FGF3_HOMSA
FGF3_GALGA
FGF3_XENLA
FGF3_DANRE
FGF5_MUSMU
FGF5_HOMSA
FGF5_GALGA
FGF5_DANRE
FGF6_DANRE
FGF6pre_GALGA
FGF6_MUSMU
FGF6_HOMSA
FGF4_DANRE
FGF4_MUSMU
FGF4_HOMSA
FGF4_GALGA
FGF4I_XENLA
FGF4II_XENLA
FGF456_CIOIN
FGF371022_CIOIN
FGFlarge_CIOIN
FGF9_GALGA
FGF9_MUSMU
FGF9_HOMSA
FGF9_XENLA
FGF20pre_GALGA
FGF20_MUSMU
FGF20_HOMS
FGF20_XENLA
FGF20B_DANRE
FGF20A_DANRE
FGF16_DANRE
FGF16_MUSMU
FGF16_HOMSA
FGF16_GALGA
E4XRB7/37-160
FGF91620_CIOIN
E4YXH3/27-160
E4YNV8/28-160
FGF11121314_CIOIN
FGF13_DANRE
FGF13_MUSMU
FGF13_HOMSA
FGF13_GALGA
FGF13_XENLA
FGF14_DANRE
FGF14_GALGA
FGF14_MUSMU
FGF14_HOMSA
FGF12_MUSMU
FGF12_HOMSA
FGF12_GALGA
FGF12_DANRE
FGF11_DANRE
FGF11_MUSMU
FGF11_HOMSA
FGF1_DANRE
FGF1_XENLA
FGF1_GALGA
FGF1_HOMSA
FGF1_MUSMU
FGF2_DANRE
FGF2_XENLA
FGF2_GALGA
FGF2_MUSMU
FGF2_HOMSA
FGFNA1_CIOIN
FGF23_DANRE
FGF23_MUSMU
FGF23_HOMSA
FGF23_GALGA
FGF19_HOMSA
FGF15_MUSMU
FGF19_GALGA
FGF19_DANRE
FGF21_MUSMU
FGF21_HOMSA
0.3
